# A strategic stakeholder approach for addressing further analysis requests in whole genome sequencing research

**DOI:** 10.1186/s40504-016-0037-3

**Published:** 2016-04-18

**Authors:** Bradley Steven O. Thornock

**Affiliations:** Philosophy Department, Saginaw Valley State University, 7400 Bay Rd. University Center, 48710 Saginaw, MI USA

## Abstract

Whole genome sequencing (WGS) can be a cost-effective and efficient means of diagnosis for some children, but it also raises a number of ethical concerns. One such concern is how researchers derive and communicate results from WGS, including future requests for further analysis of stored sequences. The purpose of this paper is to think about what is at stake, and for whom, in any solution that is developed to deal with such requests. To accomplish this task, this paper will utilize stakeholder theory, a common method used in business ethics. Several scenarios that connect stakeholder concerns and WGS will also posited and analyzed. This paper concludes by developing criteria composed of a series of questions that researchers can answer in order to more effectively address requests for further analysis of stored sequences.

Whole genome sequencing (WGS) can clearly improve diagnosis and treatment of children in some cases (Soden et al. 2014), but it also raises a number of ethical concerns. One prominent ethical issue is how to manage the incidental findings that occur whenever a child’s entire genome is sequenced (Berg et al. 2013). The American College of Medical Genetics recommended that certain findings be returned regardless of whether they were the results for which the testing was undertaken or whether parents requested those results. By contrast, the American Academy of Pediatrics recommended that only test results that would result in a change in clinical management during childhood should be reported (Green et al. 2013). Other scholars suggest establishing procedural approaches for making decisions, rather than specifying that any particular findings be returned or withheld (Koenig 2014).

One way to avoid the need to deal with most incidental findings is by only examining genes that have a high likelihood of being associated with a child’s clinical condition. As described in Saunders et al. (2012), this approach is a part of on-going research investigating the clinical utility of WGS for sick newborns in a neonatal intensive care unit. Current sequencing technology allows results to be available in less than 48 h, though the interpretation of sequencing results could take longer. In order to increase the clinical and economic efficiency of sequencing, geneticists may analyze only those portions of the genome that have a high likelihood—based on prior studies—of being associated with the particular symptoms of the newborn. Though the entire genome is sequenced, the sequence is filtered so that only those variants related to a newborn’s symptoms are fully analyzed and returned to the treating physician. This approach only generates information about specific genes and as such it is less likely than a more comprehensive analysis of the genome to reveal unsought information.

This approach will not avoid every incidental finding since there may be some incidental findings in the specific genes that are analyzed. But it will decrease the number of such findings. It will not, however, eliminate the need for difficult ethical choices. Instead, it will raise a new set of important questions regarding when and how researcher ought to communicate any remaining results of a WGS.

Every newborn that undergoes WGS will have their entire genome sequence stored. In addition, both parents’ genomes are sequenced and stored in order to investigate *de novo* mutations. While the information in these stored gene sequences may be incidental to a child’s current illness, it may yield information that is important to the child’s future health. It is, therefore, likely that, in some cases, parents or physicians will request further analysis of a child’s genome. These requests could potentially run the gamut from those intimately connected to the child’s welfare to those based on curiosity. Geneticists and pediatricians will have to decide whether every such request should be honored or, if not, to articulate criteria by which such requests should be evaluated. Answers to questions have implications for any use of WGS as a clinical tool.

The purpose of this paper is to propose limited theoretical criteria in order to think about what is at stake, and for whom, in any solution that is developed to deal with these complex questions. Because these questions extend beyond WGS or healthcare management, I will develop these criteria by using stakeholder theory—a method that is often used in business ethics to understand the issues that arise when conflicting loyalties and interest groups create management challenges.

## Stakeholder theory

Edward Freeman’s book, *Strategic Management: A Stakeholder Approach* (1984), defines a stakeholder as “any group or individual who can affect or is affected by the achievement of the organization’s objectives” (pg. 46). Stakeholders for an organization could include its customers, employees, suppliers, regulators, critics and competitors. A stakeholder approach to strategic management seeks the serious inclusion of these various stakeholders’ values and interests into the everyday workings of organizations. Within stakeholder theory, an organization “can be viewed as a set of interdependent relationships among primary stakeholders” (Hillman and Keim 2001, pg. 127), or as the place where ongoing voluntary agreements or contracts between stakeholders and an organization’s managers intertwine (Freeman and Phillips 2002). Stakeholder theorists argue that an organization’s success depends on how well it manages its relationships with these key groups (Phillips et al. 2007).

A central admonition of stakeholder theory is that managers in organizations need to attend to the interests of individuals and groups who can either assist or obstruct an organization’s objectives (Phillips et al. 2007). In other words, an organization’s success is dependent on how well it manages its relationship with the key groups that can affect its purposes (Freeman and Phillips 2002). Stakeholder theory sees management as being morally responsible to all stakeholders “because business actions harm or benefit various stakeholders, or because stakeholders have rights, or because stakeholders should be part of the processes which meaningfully affect their lives” (Gilmartin and Freeman 2002, pg. 56). Stakeholder theory does not, however, make it incumbent on managers to treat each stakeholder equally or require managers to take from one group of stakeholders and give to another (Freeman and Phillips 2002). Instead, stakeholder theory is simply stating that it is practically and ethically advantageous for a manager in an organization to take into account the groups and individuals affected or who can affect the organization’s goals and purposes (Freeman et al. 2004).

Stakeholder theorists have developed several steps for accounting for the values and preferences of stakeholders (Parmar et al. 2010). First, managers need to recognize that it is counterproductive to separate “ethical” aspects of decisions or policies from “business” aspects (Gilmartin and Freeman 2002). There is a tendency for managers to analyze actions such as acquisitions, mergers, transactions, and payments as if they are somehow devoid of any ethical or human dimensions—that they are strictly business. Stakeholder theorists consider this separation fallacious because both ethics and business are concerned with creating something of value to individuals (Freeman and Moutchnik 2013; Gilmartin and Freeman 2002). A principal task of stakeholder theory is to pragmatically and logically find ways to stitch “business” and “ethics” back together (Freeman et al. 2004). This project extends across the spectrum of business ventures, including those involved in healthcare (Gilmartin and Freeman 2002). Stakeholder theory looks to place creating value for human beings at the forefront of all business considerations (Freeman et al. 2010).

Second, managers need to determine the purpose of the firm by “articulat[ing] the shared sense of the value [an organization] create[s], and what brings its core stakeholders together” (Freeman et al. 2004, pg. 364). An organization’s purpose goes beyond just solvency and includes creating value for individuals. Each organization must have a moral vision that reflects where it has been, where it ought to go, and what choices and actions are necessary to get there (Freeman 2010).

Third, managers need to recognize which stakeholders are key to the organization’s overall purpose. These key stakeholders are those individuals and groups toward whom the organization has distinct duties such as contractual responsibilities or other “direct moral obligation toward their well-being” (Phillips et al. 2007, pg. 489).

Finally, managers need to decide how to allocate the organization’s goods and resources. This involves asking a series of questions including: how does the organization and its managers interact with stakeholders; what values, social issues, and stakeholder expectations is the organization ready to meet now; when might the organization be ready to act on a stakeholder’s desire; and how should the organization go about fulfilling a stakeholder’s desire (Freeman 2010; Minoja 2012).

These steps of stakeholder management may be especially helpful in times in which the desires of one party conflict with those of other parties or with the goals of the firm. Instead of forming a zero-sum-game logic, in which one party loses so another can gain, managers can place stakeholder desires into the larger context of the firm, so that if a desire must be denied or delayed it is in the long-term benefit of *all* stakeholders (Gilmartin and Freeman 2002). Stakeholder management can assist organizations achieve their overarching goals while still responding to specific stakeholder needs and desires.

## Stakeholder theory and genomics

It is at this level that stakeholder management may benefit research organizations conducting genomic testing. Consider the following three scenarios:

1. One year after the death of their newborn, who died despite undergoing rapid WGS, the deceased newborn’s parents request that the newborn’s sequence and their own sequences be fully analyzed, including variants for child and adult-onset diseases, with the complete results being returned to them for family planning purposes.
2. One year after undergoing rapid WGS, which was able to determine a diagnosis that saved the newborn’s life, the infant’s pediatrician requests that the rest of the stored sequence be analyzed, including variants for child and adult-onset diseases, with the complete results being returned to the doctor
3. One year after undergoing rapid WGS, which was able to determine a diagnosis that saved the newborn’s life, the infant’s pediatrician requests that certain portions of the stored sequence be analyzed and returned to the doctor in order to possibly explain some chronic symptoms the infant is currently experiencing.

Determining which request, if any, should be honored requires researchers to develop criteria that not only justify why certain requests should be fulfilled, but also why other requests were not. Stakeholder management strategies may aid in the development of such a criteria.

In the case of Soden et al. (2012), stakeholders should have input in decisions regarding the use of stored sequences. Stakeholders clearly have a continued interest in these sequences even after they have been used for their primary purpose. Many proposals for including stakeholders in decisions about how to use stored sequences propose that the decisions can be made at one point in time, usually at the outset of the study. Thus, they may ask research participants for permission to use these sequences for future research. By this view, those future research projects are an integral part of the ways in which the enterprise provides value to stakeholders. Those stakeholders need to remain both included and invested in the project.

Researchers might be tempted to separate ethics from what they deem as purely scientific or technological actions. For instance, in WGS research, it may be tempting to see ethics as an integral component at the bookends of a study, at the beginning when obtaining consent or at the end when returning results, but see other steps (sequencing, analyzing, verification, storage) as wholly technological or scientific endeavors separate from ethics. However, these actions are not devoid of ethics because they are directly related to how researchers provide value to their stakeholders. Researchers should not assume that the storage of sequences is merely a technological or pragmatic necessity devoid of ethical obligations.

Researchers need to work with stakeholders to decide what type of value they are trying to build and for whom they are building it. This involves asking two fundamental questions: first, what is the purpose of gene sequencing; second, for whose benefit was the sequencing undertaken. Answering these questions allows researchers to “articulate the shared sense of value [they] create, and what brings [their] core stakeholders together” (Freeman et al. 2004, pg. 364).

Saunders et al. (2012) describe the purpose of the rapid WGS research as aiding in the diagnosis of acutely ill newborns. Specifically, the aim of sequencing is to improve clinical outcomes for some newborns and to do so in a timely and cost-effective manner. Therefore, narrowing the analysis of sequences to only those variants linked with the infant’s symptoms is not just a tricky means of avoiding incidental findings. It is a necessary step to allow such testing to meet its primary goals in a timely and cost-effective way. The aim of this research is also deeply humanistic: by trying to find better ways to treat a critically ill newborn, researchers are providing a glimmer of hope in an otherwise desperate situation. But, the storage of sequences is done for more than just this primary goal and may aid in other research goals. Stakeholders must buy into and support those other goals or else they may become alienated from the entire project.

A next step in stakeholder management is to identify the individuals and groups who stand to benefit from WGS. There are numerous stakeholders for this rapid WGS research and include regulators, lab personal and hospital staff, public health authorities, device manufacturers and others. But the key stakeholders, in the case of Soden et al. (2012), are the newborn, the parents and the treating physician. These researchers have a direct duty or moral obligation toward each of these primary stakeholders because these stakeholders are intimately tied with the diagnostic and clinical aims of rapid WGS research. Because the gene sequences are being retained, these primary stakeholders continue to have a vested interest in the way the sequences are used even after the original obligations have been met.

Research funders, such as the NIH, may or may not be included in these key stakeholders depending on the stipulations of the research protocol and grant. In the case of Soden et al. (2012), the research protocol only covered the release of WGS information that was pertinent to the immediate care of an ill neonate. As such, the analysis of stored sequences is supererogatory and may not be funded by the original grant. In such instances the parents themselves are going to likely pay for the additional analysis without the aid of third parties. Researchers could, of course, amend their existing grants or seek new funding to pay for the analysis of stored genomes in which case they should ensure that the requirements of an external funder do not overwhelm the needs of other key stakeholders.

After articulating the purpose of the research and identifying primary stakeholders, researchers need to establish certain procedures that can guide transactions between these primary stakeholders and themselves. Researchers need to be clear about the answers to a number of questions. How they will interact with these primary stakeholders on an ongoing basis? How should they evaluate the requests of primary stakeholder’s to use the genomic data? If they want to honor stakeholders’ requests, how should the researchers go about fulfilling those requests without detracting from other goals? (Minoja 2012; Freeman 2010). Answers to these questions will allow researchers to build a strategy that will allow research to continue toward its stated purposes while still allowing them to respond to new requests made by certain primary research stakeholders.

After such an exercise, researchers should be better equipped to address the scenarios presented earlier. For the rapid WGS, researchers may be justified in dismissing a request made by solely by parents (e.g., scenario 1). Though parents are primary stakeholders, rapid WGS is being investigated as a clinical tool, not as a direct-to-consumer product. Researchers have yet to establish procedures for working directly with parents, making this type of expansion premature and inappropriate.

Since the protocol for rapid WGS was built around using the treating physician as a liaison between the researchers and the parents, researchers are better suited to follow a similar format to address requests for further analysis. In addition, since rapid WGS is specifically being investigated as an aid in diagnosis, physician requests that exceed this aim (e.g., scenario 2) should also be rejected. Similar to requests coming directly from parents, the research protocols for rapid WGS are simply not suited for returning such a wide range of genomic results. Therefore, requests for further analysis made by a treating physician for a diagnostic purpose (e.g., scenario 3) are those most fitting for this rapid WGS research protocol.

In deciding to fulfill some requests while ignoring others, researchers should not pit the desires of one group of stakeholders over those of other groups. Instead, researchers should aim to find ways that can harmonize primary stakeholders’ desires with the overall purpose of the research. For example, they might encourage parents to enlist the help of a physician to act as a liaison between them and the researchers. In addition, researchers may not be able to fulfill certain requests right now, such as using WGS for family planning; however, researchers can still work with primary stakeholders to determine a timetable in which their request for family planning analysis could be addressed. Finally, researchers need to be transparent as possible regarding why certain requests are being honors while other are not. Openly discussing how these decisions are made can help to build trust and avoid alienation.

The criteria for deciding which requests for further analysis ought to be considered should grow organically and pragmatically from current research practice. In this way, researchers and research stakeholders work together toward the benefit of all. Over taxing researchers and straining protocols not only puts current research efforts at risk, it also seriously undermines the researchers’ ability to fulfill any additional requests for further analysis.

## Conclusions

Drawing from stakeholder theory, this paper developed a series of questions that researchers can ask when determining whether to fulfill the request of a primary research stakeholder. These questions include: what is the purpose of the research, for whose benefit was the research undertaken, does a primary stakeholder’s request fit within the goals of the research, when might the researchers be ready to act on the request, and how should the researchers go about fulfilling the request. While this paper applied these questions specifically to rapid WGS research, they can assist researchers conducting a variety of different research protocols address the changing needs of stakeholders. Establishing such a criteria before requests are made will be a great asset to researchers who may face evolving needs and desires of research stakeholders.
